# Fischer–Tropsch Synthesis for the Production of Sustainable Aviation Fuel: Formation of Tertiary Amines from Ammonia Contaminants

**DOI:** 10.1021/acsomega.4c03734

**Published:** 2024-07-10

**Authors:** Robert L. C. Voeten, Floran Hendriks, G. Leendert Bezemer

**Affiliations:** Energy Transition Campus Amsterdam, Shell Global Solutions International B.V., Grasweg 31, 1031 HW Amsterdam, The Netherlands

## Abstract

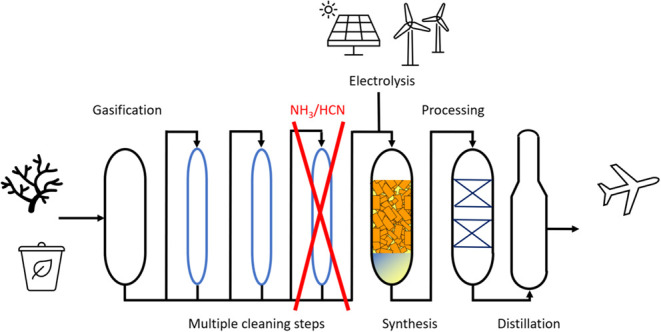

Fischer–Tropsch synthesis combined with product workup is a promising route toward synthetic aviation fuel from renewable hydrogen and carbon sources like biomass, CO_2_, and waste. Cost savings can be achieved by reducing the number of gas treatment steps in new plants, but the consequence of contaminants in the feed needs investigation. While feeding 2.6 ppmV ammonia to a Fischer–Tropsch reactor, it was shown that ammonia was predominantly chemically converted into organic amines, with most nitrogen found in the water phase (89%), followed by heavy wax (7%) and light wax (1%). The concentration difference between water and light wax was shown to be due to the post-condensation separation of amines on polarity. Amines up to a chain length of 120 were detected in the heavy wax with MALDI-FT-ICR-MS, which, in combination with the high nitrogen content, suggests that amines have a similar chain growth probability compared to the main hydrocarbon products. Detailed product analysis with three independent analytical techniques showed that tertiary *N*,*N*-dimethylalkylamines were by far the most abundant amine class. This suggests that ammonia is decomposed on the cobalt surface and, potentially as a dimethylamine fragment, incorporated in the growing chain. Further evidence was obtained from the abundance of trimethylamine and from the reconciled nitrogen product analysis up to C100, which showed that the amine product distribution followed from naphtha onward the same ASF kinetics as alkanes and oxygenates while being distinctively different from the alkene distribution. The presented findings provide further avenues for studies of the Fischer–Tropsch reaction mechanism and indicate the opportunity of cost saving on gas treatment, while further validation is required to assess the impact on hydrocracking and product quality.

## Introduction

The Fischer–Tropsch (FT) reaction involves the conversion of CO and H_2_ into hydrocarbons via surface polymerization on supported cobalt or iron catalysts. It is a proven commercial process that is applied to produce chemicals and fuels from non-crude feedstocks. Nowadays, the FT process receives a lot of attention from both industry and academia as one of the mature options to produce synthetic aviation fuel (SAF), which even has superior quality, resulting in lowered contrail formation.^1−7^ Biomass and waste streams can be used after gasification, while CO_2_ needs conversion into CO with the reverse water gas shift reaction. The ReFuelEU directive mandates from 2025 onward increasing minimum amounts of both sustainable and synthetic aviation fuel but mentions that currently estimated costs are three to six times higher than the market price of conventional aviation fuel, a range which is in line with academic studies.^4,8−10^ Sustainable fuels from syngas after biomass gasification require less green hydrogen compared to CO_2_-based routes and are more affordable, while combinations with renewable hydrogen can retain up to 96% of carbon present in the feedstock.^11^

**Figure 1:**
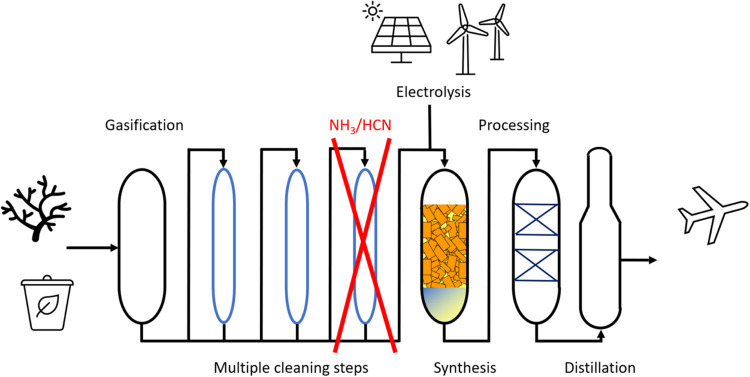
Simplified process line-up indicating how carbon input from waste and biomass is processed via gasification. Multiple cleaning steps are followed to polish the CO/H_2_ mixture, which is reacted by Fischer–Tropsch synthesis and hydroprocessing into sustainable aviation fuel (SAF). Cost reduction of the line-up is obtained by reducing gas treating steps but results in the presence of NH_3_/HCN at ppmV concentrations in the gas mixture toward the catalytic reactor.

Synthesis gas produced by biomass gasification can contain dust and soot, tar-like impurities, and gaseous impurities like NH_3_ and HCN.^12^ Impurities are removed by a series of wet and dry treatment steps, and capital cost related to gas cleaning has been estimated to be more than 10% for smaller plants.^13^ Target concentrations of NH_3_ and HCN below 0.05 ppmV have been proposed for cleaned synthesis gas.^14^ Line-up simplification and substantial cost reduction can be obtained if higher amounts of N can be accepted in the feed toward an FT reactor.^15^ In Figure 1a, a simplified line-up to produce SAF from biomass or biomass and renewable hydrogen is depicted, indicating that the potential impact on both the FT section and the units further downstream needs to be considered before allowing less treatment. The catalytic impact of HCN and NH_3_ on the performance of FT catalysts has been evaluated in various studies. Contrary to iron-based catalysts, where no impact has been observed,^16−18^ on cobalt-based systems, which are the catalysts of choice due to excellent product selectivity toward SAF, various impacts have been reported.^18−24^ Borg et al. reported no catalytic impact of 4.2 ppmV ammonia with a rhenium-promoted catalyst,^22^ whereas studies of the Davis group showed a drop in CO conversion of around 40% while working with 10–1000 ppmV NH_3_ on alumina-, silica-, and titania-supported catalysts.^23,24^ Similarly, the group of Khodakov reported more than 50% reduction of activity while working with 1500 and 2500 ppmV co-feeding of ammonia and acetonitrile.^18^ On a fundamental level, Niemantsverdriet et al. showed that NH_3_ adsorption was not blocked by the presence of CO_ad_ and H_2ad_ and that dissociated NH_*x*_ species had a higher stability on the cobalt surface and were most likely responsible for the observed effect on catalyst activity.^19,20^

Besides understanding the catalytic impact on the FT section, it is vital to know the molecular speciation of nitrogenates in the Fischer–Tropsch effluent, as this determines which downstream processing units may be impacted. None of the studies cited above reported on the nitrogen speciation downstream the FT reactor, likely related to the difficulty in quantifying and characterizing nitrogenates at low concentrations. Working at much higher NH_3_ concentrations of 2–10%, there are several studies that include product analysis.^25−32^ Selective formation of acetonitrile was reported over FeRh alloys,^25^ while mixtures of amines or mixtures of amines, nitriles, and amides were found with Fe catalysts^26,27,30,31^ and mixtures of amines and nitriles with a Co_4_Mn_1_K_0.1_ catalyst.^28,29^ Co-feeding 20% dimethylamine over iron catalyst mixtures, tertiary alkylamines were obtained.^32^ The longest nitrogenate length reported in the studies varied from 7,^28^ 9,^31^ 15^30^ to 19^26,32^ carbon atoms, and product analysis was in all cases reported for a single outlet stream only. No information on the distribution of nitrogenates over the various outlet streams was reported. The present paper will demonstrate the presence of nitrogenates in three outlets: water, light wax, and heavy wax. This is done while studying a fixed bed titania-supported cobalt catalyst and using only 2.6 ppmV ammonia in the gas phase, a concentration relevant for nontreated biomass-derived synthesis gas.^12^ Detailed product analysis is applied that indicates that tertiary amines are formed predominantly, which have a similar chain growth probability as the main hydrocarbon product.

## Experimental Section

### Fischer–Tropsch Synthesis

The Fischer–Tropsch synthesis reaction was carried out in a fixed bed reactor with an inner diameter of 40 mm. The catalyst consisted of 25 wt % cobalt supported on P25 titania and was made by extrusion as described earlier.^33^ A thermowell was loaded in the middle of the catalyst bed, allowing temperature measurement along the axial direction of the catalyst bed. Prior to the start of the reaction, the catalyst was reduced with hydrogen in situ at 260 °C while keeping water concentration below 3000 ppmV.^34^ The reduction degree was above 90%, and the cobalt surface area amounted to 4.8 m2/g. After the reduction, the reactor temperature was lowered to 160 °C, and reactor pressure increased to 60 bar. After the introduction of CO and H_2_, the temperature was increased to reach an average bed temperature of 231 °C. The reactor was operated in recycle mode with inlet gas concentrations of 21% N_2_, 49% H_2_, and 30% CO. The catalytic data were reported after 3 weeks of operation. Ammonia was constantly added to the gas heater using a diluted ammonium hydroxide solution. The amount of ammonia added per hour was converted into a gas flow and divided by the total gas flow to obtain an ammonia gas phase concentration, which amounted to 2.6 ppmV. The ingoing amount of nitrogen was determined by multiplication of the ammonium concentration as obtained with ion chromatography and the weight of the dosed liquid. The off-gas stream was analyzed by passing the gas through a series of scrubbers filled with acidified demineralized water to capture potential ammonia and light amines. Liquid from scrubbers was analyzed on total nitrogen content with chemiluminescence. The heavy wax, light wax, and water products were obtained from high-pressure separators operating at respectively 183 and 16 °C. The total nitrogen content in each of the streams was measured with chemiluminescence, which resulted after multiplication with the weight of the streams in the outgoing amounts of chemically bonded nitrogen.

### Product Distribution

The composition of the various gas streams was analyzed online using a Siemens Maxum II Gas Chromatograph (GC) with thermal conductivity and flame ionization detectors (FIDs). Light wax was analyzed with an Agilent 6890 GC, and the heavy wax samples were analyzed using a Trace GC-Ultra from Thermo Scientific; both instruments were equipped with an FID. For light wax, GC × GC was used with a capillary column with the dimethylpolysiloxane stationary phase for boiling point separation and a capillary column with the chemically bonded cross-linked 50% polysilphenylene siloxane stationary phase for polarity separation. Heavy wax separation was done with the same stationary phase and was used to quantify hydrocarbons up to C100. Full carbon distribution was obtained by combining online and offline hydrocarbon analysis results with the mass flows of each stream. Discrimination between paraffins, olefins, and oxygenates was possible in the light wax stream, whereas in the heavy wax stream, combined results per carbon number were obtained. Consequently, the obtained carbon distribution extended to C22 (oxygenates), C30 (olefins), and C100 for amines and combined hydrocarbons. The quantitative concentration of alcohols up to undecanol in the water phase was obtained by GC analysis using 2-ethylbutanol as an internal standard, a capillary column, and FID detection. The concentrations were calculated from the relative peak area versus the internal standard while correcting for differences in FID response with theoretical response factors.

### Materials

Purified water (18.2 MΩ cm) was obtained from a Milli-Q purification system (Merck Millipore, MA). Methanol (MeOH; MS-grade), formic acid (FA; 98–100%), and acetonitrile (ACN; MS-grade) were obtained from Merck (Darmstadt, Germany). Toluene (Spectronorm) was obtained from VWR, and 2,5-dihydroxybenzoic acid (DHB; >98%) was obtained from TCI. All amine standards (e.g., methylamine, ethylamine, *n*-propylamine, etc.; > 98%) were purchased from Thermo Scientific except for docosan-1-amine and *N,N*-dimethyldodecylamine (>96%), which were acquired from Apollo Scientific and TCI, respectively. The eluent cartridge for methanesulfonic acid (MSA) was obtained from Dionex.

### Sample Preparation

All water samples were diluted (10–100× depending on the analysis) in water prior to injection except for using solid-phase microextraction (SPME) GC mass spectrometry (MS) analysis. For MS analysis, 0.1 v/v% FA was added to the solutions. Light wax samples were diluted in 50/50 MeOH/toluene containing 0.1% (v/v) FA. The heavy wax sample was ground to a fine powder, and subsequently, (I) 4 mg was dissolved in 1 mL of toluene at 80 °C, followed by a 1:1 (v/v) dilution with MeOH, yielding a final concentration of 2 mg/mL, and addition of 0.1% FA and (II) mixed with 10 mg of DHB (1:1 m/m), respectively, for electrospray ionization time-of-flight (ESI-TOF)MS and matrix-assisted laser desorption ionization (MALDI) Fourier transform ion cyclotron resonance (FT-ICR-) MS analysis.

### Instrumentation and Methods

Comprehensive details on experimental information, including systems and methods, are provided in the Supporting Information. Total nitrogen contents were determined using chemiluminescence with a Thermo Analyzer TN3000 for water, light wax, and heavy wax samples (5 mg of the sample). For ion chromatography with conductivity detection (IC-CD), a Thermo Scientific IC-5000 system equipped with a Thermo Scientific CS10 column (2 mm × 250 mm) using 20 mM methanesulfonic acid (1 mL/min) as the eluent was employed for characterizing and quantifying amines up to C3. Water samples were diluted 10× in water before analysis via flow-injection analysis (FIA; *V*_inj_ = 10 μL) with an Agilent 1290 Infinity II coupled to a single quadrupole mass spectrometer (SQ-MS; Agilent 6135 MSD XT) for detecting amines up to C11. Characterization of the C5 to C80 range in water, light wax, and heavy wax samples was conducted using a Synapt-G2 Si TOFMS system (Waters) and up to C120 using a high-resolution 12T Bruker solariX XR FT-ICR-MS (Bruker Daltonics, Bremen, Germany). Liquid chromatography (LC) separations were carried out on a Waters Acquity UPLC system using a Kinetex 1.7 μm BEH C8 column (100 mm × 2.1 mm) with a water–ACN gradient. GC was performed using SPME with a multipurpose PMDS/DVB/CWR fiber at a temperature of 60 °C. The GC operated in splitless mode, and the separation was on an SPSil 5CB column (50 m × 0.32 mm × 1.2 μm). All LC-MS systems were equipped with an ESI source in positive mode. The GC-MS system used an electron impact (EI) source. Direct infusion was performed with 10 μL/min flow rates unless otherwise specified.

### Data Processing

All IC-CD results were processed using Chromeleon 7.2.9 (Thermo Scientific) and all FIA-SQ-MS with OpenLabCDS 2.7.1 (Agilent Technologies). For standard addition calculations, Excel (Microsoft 365 Version 2208 64-bit) was used. LC-ESI-MS(/MS) data were recorded and processed with MassLynx V4.2 (Waters). The FT-ICR-MS spectra were processed using DataAnalysis 5.3 software (Bruker Daltonics), exported as csv files, and imported into Composer software (Sierra Analytics) for molecular formula annotation. These outputs were transferred to Excel for visualization of the amine distributions. The hydrophobic/lipophilic balance (HLB) was calculated using the Davies method, which stipulates values of 1.9 and 9.4 for the OH and N functional groups, respectively, and −0.48 per CH_*x*_ moiety.^35,36^

## Results and Discussion

**Figure 2:**
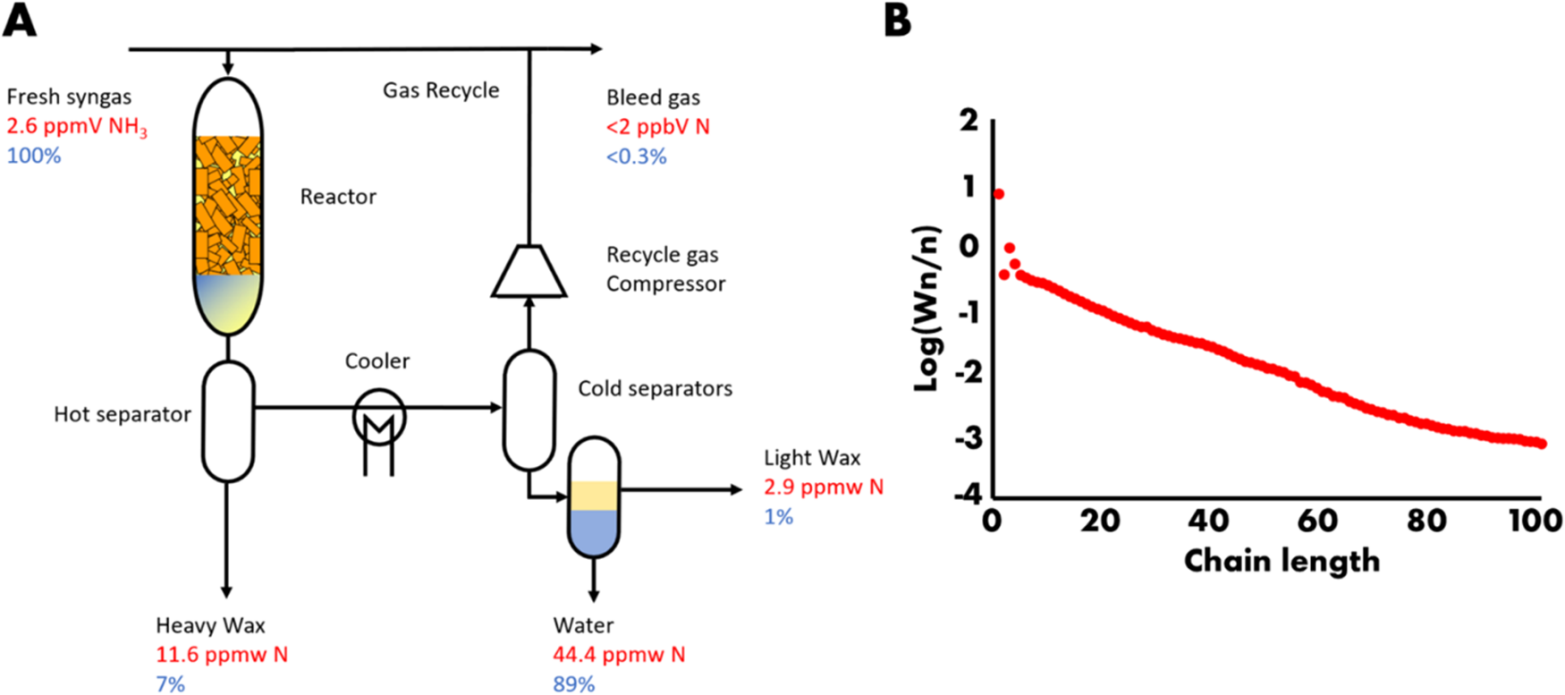
(A) Line-up of process units and separators during Fischer–Tropsch testing. The nitrogen concentrations in the inlet stream and the various outlet streams are indicated in red. The relative N amounts versus the intake are provided in blue, indicating that the majority resides in the water stream. (B) Full product distribution as the ASF plot from combined off-gas, light wax, and heavy wax analysis showcasing that wax heaviness extended beyond C100.

The Co/TiO_2_ catalyst was loaded in a fixed bed reactor, and after in situ reduction, the catalytic performance was evaluated in the presence of 2.6 ppmV ammonia (Figure 2A). The reactor was operated in recycle mode at 60 bar. The molecular nitrogen balance was 101%, and the total mass balance was 99%. The catalyst activity was 225 g·l_cat_^–1^·h^–1^ with 1% CO_2_ selectivity and a liquid product selectivity of (C_5+_) of 88 wt %. The full product distribution (Figure 2B) was measured up to C100, with 10 wt % product being heavier than C100. The catalyst displayed Anderson–Schulz–Flory (ASF) kinetics from C5 onward with the typical overshoot of methane and undershoot of ethane/ethene that characterizes metallic cobalt systems.^37,38^ The chain growth probability increased with product heaviness to above 0.95, as visible from the bend in the product distribution above C70. Deviations from a single α product distribution have been observed earlier and have been attributed to olefin readsorption or gradients in process conditions.^39−42^ All product streams were analyzed on chemical nitrogen content. The off-gas contained <2 ppbV nitrogen, indicating that there was no buildup of ammonia or lighter amines in the gas loop during the catalytic test. The heavy wax stream from the high-temperature separator contained 11.6 ppmw chemically bonded nitrogen, equivalent to 7% of the amount dosed. The condensed light wax and water streams contained, respectively, 2.6 and 44.4 ppmw nitrogen, amounting to 89 and 1% of the intake, respectively. 3% nitrogen not accounted for is ascribed to imprecisions in the nitrogen measurements. In the acidic process, water–ammonia is present as ammonium ions and the ammonium content amounted to 23 ppmw, indicating that 60% was chemically bonded to carbon and (vide supra) present as alkylamines. The low concentration of nitrogen in the light wax versus the water and heavy wax streams can be rationalized by the hydrophilicity of the amines (vide infra). Alkylamines have an intermediate position compared to alcohols and soaps and are predicted to be hydrophilic up to C12 and water-dispersible up to C21. Dissolution of alkylamines in the water phase in the cold separator reduces the nitrogen concentration in the light wax as compared to the heavy wax.

### Amine Distributions

The amine distributions in the three effluent phases were determined with a suite of analytical methods and will be discussed in order of decreasing N concentration, starting with water.

#### Water

**Figure 3:**
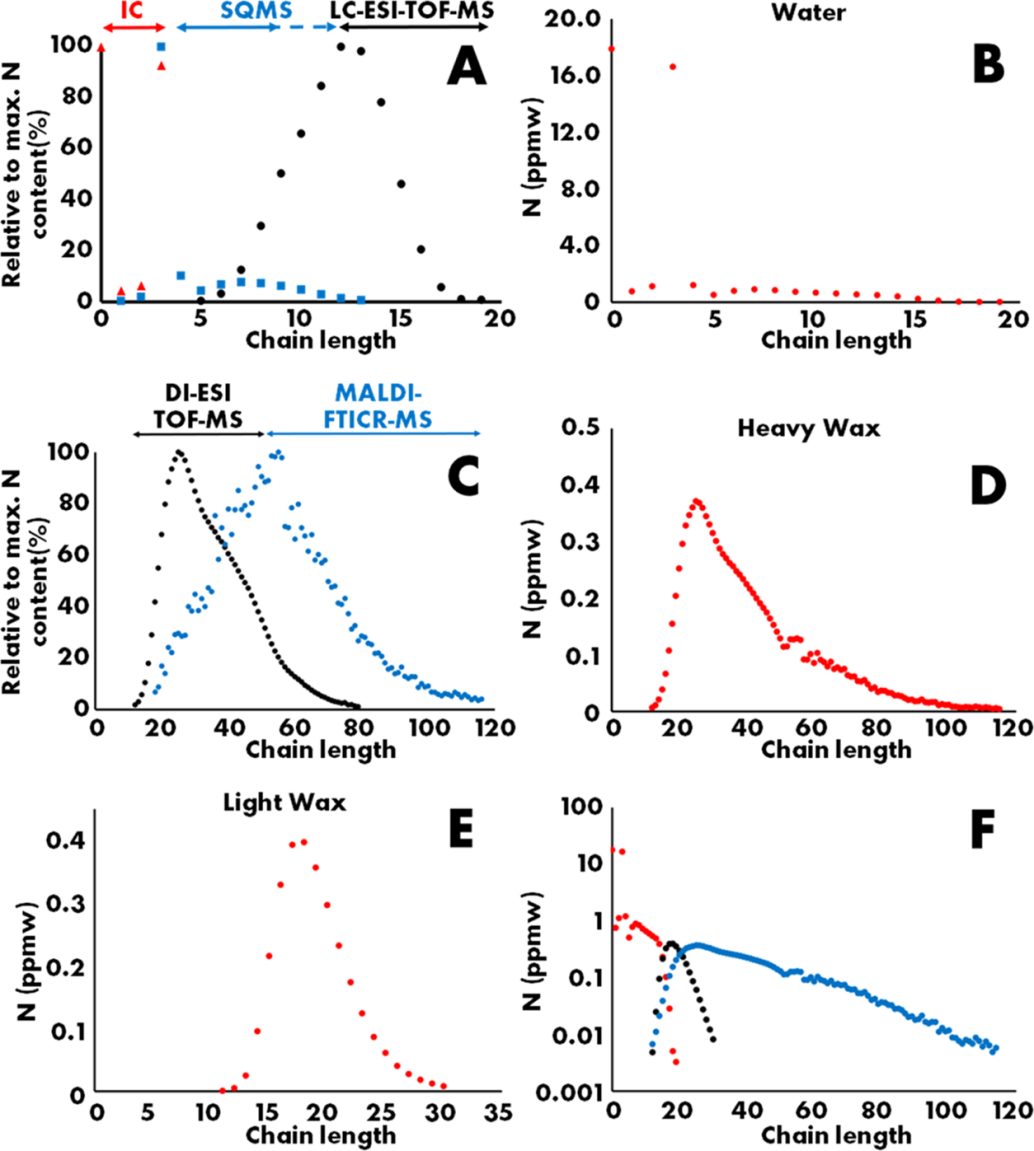
(A) Relative concentration in the water sample normalized on N content plotted versus carbon number from ion chromatography (red), single quadrupole mass spectrometer (blue), and LC-ESI-TOFMS (black) with the domain indicated where each respective technique was used for quantification. (B) Concentration of ammonia and amines versus carbon chain length. (C) Relative amine concentrations in heavy wax plotted versus chain length from DI-ESI-TOFMS (black) and MALDI-FT-ICR-MS (blue). (D) Amine concentration in heavy wax using integrated ESI and MALDI data. (E) Amine content distribution in the light wax sample when dissolved in 50/50 toluene/methanol containing 0.1% formic acid. (F) Ammonia and amine concentrations in water (red), light wax (black), and heavy wax (blue) versus chain length.

The sample was directly infused and analyzed with ESI-TOFMS after 100× dilution. The resulting mass spectrum and corresponding amine distributions are provided in Figure S1a,b, respectively. The spectrum reveals an *m*/*z* distribution spanning *m*/*z* 88.113 to 284.332 with increments of *m*/*z* 14.0156 and is attributed to C5–C19 protonated amines with C12–13 observed as most intense and C5 and C19 as least intense (Figure 3A black trace), which agrees with previous observations regarding amine formation when ammonia was co-fed to the FT process.^26,28^ The Fischer–Tropsch reaction exhibits product selectivity toward a homological range of hydrocarbons where the chain growth probability determines product heaviness (Figure 2B). Consequently, as a similar reaction pathway has been proposed for nitrogenates,^26,28^ the decreasing concentration of heavier amines is expected next to the substantial presence of C1–C4 amines. Hence, to complement the ESI-TOFMS analysis and to increase sensitivity toward these smaller analytes, IC-CD and FIA-sQ-MS analyses were performed on the same sample. IC analysis, employing suppressed conductivity detection, was conducted on a 20-fold diluted sample. The results, also presented in Figure 3A (red trace), reveal a prominent peak corresponding to C0 (ammonia; 100%), relatively weak signals for C1- and C2-related amines (∼5%; relative to C0), and a substantial signal for the C3 amine (∼90%). These observations are corroborated by FIA-SQ-MS (Figure 3A blue trace), which also indicates low levels of C1 and C2 (∼1 to 3%) and a notable presence of C3 (100%).

The IC results could be calibrated with standards up to C3 amine and provide the first basis for quantification. The Q-MS results were considered satisfactory, particularly in describing the low mass distribution of the alkylamines, and were quantified with C1, C2, C3, C6, and C8 amine standards. The ESI-TOFMS showed a higher sensitivity toward higher alkylamine masses in the water fraction. Hence, the data were combined in the following way: the quantitative IC method was used for ammonia and amines up to C3. Subsequently, the SQ-MS data were used up from C4–C9, and the C10–C13 concentrations were extrapolated from the chain growth factor between C7 and C9 in SQ-MS, with, finally, the C14–C19 concentrations obtained from the ESI-TOFMS data from peak maximum onward. Consolidated data in ppmw N are provided in Table S1 and presented in Figure 3B. The high concentrations of ammonia and C3 amine are, respectively, attributed to unconverted ammonia and the presence of trimethylamine (vide infra).

#### Heavy Wax

The presence of C1–C19 amines in the water phase suggested that small amounts of long amines would be present in the heavy wax stream, of which the total nitrogen concentration was 11.6 ppmw. The heavy wax sample was dissolved in toluene/MeOH/FA (50/50/0.1) and directly infused into the ESI-TOFMS system, yielding the mass spectrum shown in Figure S2. A predominant mass distribution ranging from *m*/*z* 256 to approximately 1000, with mass increments of *m*/*z* 14.0156, is observed. Although smaller masses are also detected, their prominence is less. The chemical formula assignments correspond to C12–80 amines, with the apex at C25 and a shoulder between C30 and C55 on the main distribution (Figure 3C; black). The observed amine distribution from ESI-TOFMS is compared to the paraffin distribution obtained with GC-FID (Figure S3) and shows a similar distribution profile, particularly at low carbon numbers (C13–C30). At intermediate carbon numbers (C30–45), deviations are observed. At high carbon numbers (>C50), the alkylamine intensities diminish faster compared to the paraffins, which can indicate poor extraction of long-chain amines into the toluene phase. To avoid problems with solubilizing wax-like amines, direct analysis of the solid heavy wax (complemented with dihydroxybenzoic acid to facilitate ionization) was done using MALDI (Figure S4). The MALDI-FT-ICR-MS was conducted with the MS parameters set to enhance sensitivity toward larger masses, yielding alkylamine distribution with a maximum at approximately C55 (Figure 3C; blue). The overall integrated distribution after normalization of the response at C50 spans from approximately C20 to nearly C120 alkylamines (Figure 3D). Once combined with the respective molecular weights, it could be established that the total amine concentration in the heavy wax was almost 500 ppmw (of which 11.6 ppmw nitrogen); see Table S3, showing that most of the product consists of hydrocarbons. Additional experiments revealed alkylamines up to approximately C160, of which the concentrations reach down to the ppt level (omitted), which has not been reported before. However, a drawback of MALDI-FT-ICR-MS lies in the challenging quantification particularly concerning inhomogeneous samples and variable ion suppression per sampling moment, hence allowing only indicative concentration profiles based on total nitrogen correlation.

#### Light Wax

So far, the amines partitioning across the water and heavy wax phases have been established and suggest that alkylamines should also partition into the light wax phase, which is supported by the HLB scale, the determined nitrogen content in the light wax, and would harmonize the amine distribution within the C14–C22 range. To determine the amine distribution in the light wax fraction, the sample was first subjected to liquid–liquid extraction with water and methanol (1:1 ratio). The water phase was subsequently directly infused into the ESI-TOFMS system. The obtained spectrum contained an *m*/*z* distribution corresponding to C12–C25 (Figure S5). As longer alkyl chains exhibit poorer solubility in polar solvents due to decreased polarity, the sample was also separately infused after dissolving in toluene/MeOH/FA (50/50/0.1 v/v%). The obtained spectrum contained masses attributed to C11–C30 amines, which further supported the validity of the HLB scale toward amine phase partitioning. Subsequently, we acquired the SQ-MS spectra of the light wax. Results shown in Figure S6 display a discernible CH_2_ distribution, with its apex at *m*/*z* 258.3, which is a deviation of 16 *m*/*z* from the alkylamine series and indicative of oxidation during the storage of a few weeks. Alkylamines have been reported to be susceptible to oxidation, with autocatalytic oxidation taking place at room temperature.^43,44^ Notably, Beckwith et al. demonstrated the facile auto-oxidation of tertiary amines in nonpolar solvents, while linear and secondary amines remained stable.^44^ Additionally, they highlighted that even small amounts of water substantially reduced the rate of oxidation. These observations align with the present study, where no evidence of oxidized amines is observed in aqueous samples, but severe amine auto-oxidation occurs in a hydrocarbon matrix. Quantitation of the concentration of alkylamines in the sample was based on the chemiluminescence-determined total nitrogen content (2.9 ppmw N) with concentrations of individual alkylamines calculated by distributing nitrogen across observed masses based on the determined intensity with ESI-TOFMS. Results provided in Figure 3E indicate a relatively sharp concentration profile peaking around 0.4 ppmw N for C17 and C18. After combination with molecular weights, it was established that the total amine concentration amounted to 58 ppmw (Table S3).

Consolidated results with nitrogen concentrations in water, heavy wax, and light wax are plotted versus carbon chain length in Figure 3F. The nitrogen concentrations are shown on a logarithmic scale, and the decrease in concentration with chain length resembles ASF kinetics. However, between C10 and C30, speciation is observed over the various phases, and integration of the concentration with the mass flows is required to obtain the integrated amine product distributions, which will be further discussed in Figure 5, after an intermezzo on the power of the HLB scale and the molecular nature of the amine products.

### Hydrophile–Lipophile Balance Describing Partitioning of Amines and Alcohols

**Figure 4:**
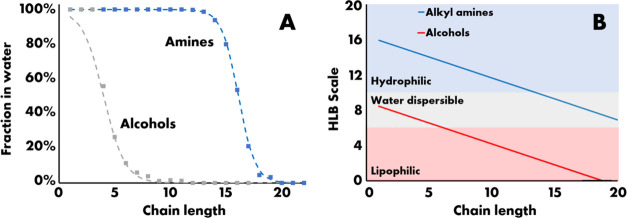
(A) Fraction of alcohols and amines present in the water phase as a function of chain length. The partitioning was also modeled using the hydrophilic/lipophilic balance for both compounds with, respectively, 6.8 and 8.7 for equal partitioning. (B) Hydrophobic/lipophilic balance (HLB) for alkylamines and alcohols plotted versus the carbon chain length.

The concentrations of alcohols and amines in light wax and water were combined with the flow rates of both streams to calculate the partitioning as a function of chain length. For amines in water and light wax, the results, as depicted in Figure 3B,E, were used. For the alcohols, we used GC × GC analysis data from the light wax and GC-FID results for the water. By combining the mass flows of the two streams with the concentrations, the partitioning in the water and hydrocarbon phases was calculated. Results are presented in Figure 4A, where the partitioning between the water and the light wax is plotted versus the carbon chain length. The partitioning of alcohols and amines is dissimilar due to the large difference in hydrophilicity, which can clearly be seen from the calculated hydrophilic/lipophilic balance as a function of carbon chain length (Figure 4B). For the amines around C15, equal partitioning in water and light wax is obtained, which corresponds to an HLB of around 9. The water-to-oil phase transition is within 8 carbon fragments. The calculated HLB could be used to describe the observed partitioning between water and light wax using 8.7 as the center point. For the alcohols in the light wax, results were obtained from butanol onward. For butanol and pentanol, the fractions in water were 61 and 31%, respectively, reducing to less than 1% for C11. Like amines, the partitioning between the phases was calculated from the molecular HLB with a value of 6.8 as the center point. The HLB corresponding to equal partitioning is for both chemicals close to 8, which is in the middle of the water-dispersible zone. A slight deviation is ascribed to differences between mixtures used here versus pure water and oil. The good correspondence between trends in the partitioning of both alcohols and amines with the HLB gives confidence about the validity of the approach and provides the explanation for the relatively low nitrogen content in the light wax versus water and heavy wax. In the full modeling of the nitrogen product distribution, the modeled partitioning, as shown in Figure S7, will be used.

### Chemical Nature of the Amines

**Figure 5:**
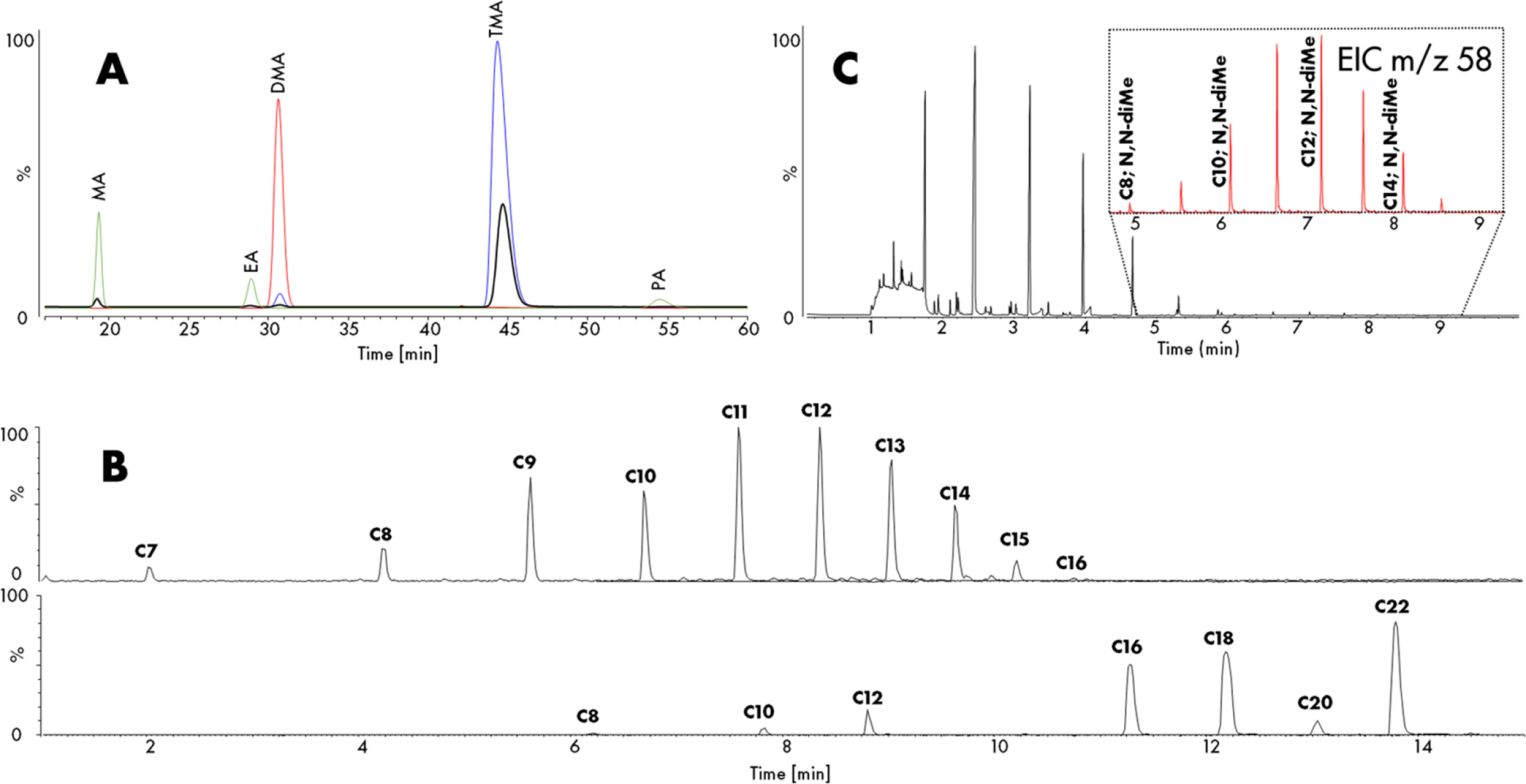
Evidence for the presence of tertiary amines from (A) overlay of ion chromatograms of the water sample in black, methylamine, ethylamine, and n-propylamine in green, dimethylamine in red, and trimethylamine in blue and (B) combined extracted ion chromatograms corresponding to (top) the masses of C7–C16 amines of the water sample and (bottom) a reference mixture containing linear *n*-alkylamines ranging C8–22 (bottom). (C) Chromatogram from an SPME-GC-MS analysis on the water sample indicating the presence of C8–C15 tertiary amines (inset: EIC of *m*/*z* 58) in the region between 5 and 9 min.

In the initial quantification method for ion chromatography on the water sample, methylamine (MA), ethylamine (EA), and n-propylamine (PA) were utilized as standards. The results (Figure 5A, green) indicated that MA and EA were eluted at 19.28 and 28.86 min, respectively, and PA was detected at 54.93 min. In our water sample (black), PA was scarcely detected, and two unidentified peaks appeared at 30.74 and 44.68 min, suggesting the presence of isomeric components. To investigate this hypothesis, dimethylamine (DMA; pink) and trimethylamine (TMA; blue) were measured, and the resulting chromatograms in Figure 5A were overlaid with the water sample (black). The conclusion from this experiment is that the predominant form of C3 amine in the water sample is TMA and that among the C2 amines, the secondary amine is more prevalent. Using external calibration, the concentrations of MA (1.68 ppm), DMA (2.57 ppm), and TMA (69.60 ppm) were determined. For comparison with total nitrogen content from chemiluminescence measurements, the nitrogen content from IC-CD measurements was established as 36.1 ppmw N (Table S1).

The presence of nonprimary amines, as revealed by IC-CD analysis, raises the question of how the chain growth would propagate. Hence, subsequent analyses were conducted to elucidate the structure of longer amines using LC-ESI-MS and SPME-GC-MS. Reversed-phase LC-ESI-TOFMS analysis was performed on the water sample and an alkylamine reference mixture. The resulting extracted ion chromatograms (EICs) for C7 up to C22 are presented in Figure 5B, top and bottom, respectively. Evidently, the retention behavior of amines with the same carbon number differs significantly when comparing the sample and reference mix. A more compact structure in the sample amines likely leads to reduced interaction with the C_8_ alkyl chains of the stationary phase, suggesting a nonlinear nature, consistent with IC-CD observations on smaller amines. These findings were further supported by SPME-GC-MS, performed by immersing the SPME fiber in the water phase. The resulting chromatogram in Figure 5C displayed prominent signals of C2–C9 alcohols, ketones, and up to C10 acids, eluting within a 7 min time frame from the GC column. At longer elution times, amine peaks were observed, spanning approximately C8–C15. Electron impact (EI) MS was used to study the molecular structure. With EI, *n*-alkylamines exhibit preferential cleavage at the carbon–carbon bond next to the N atom, resulting in the formation of the relatively stable CH_2_–NH_2_^+^ ion with *m*/*z* = 30, accompanied by CH_2_ increments (*m*/*z* 44, 58, etc.). Tertiary *N*,*N*-dimethylalkylamines undergo similar fragmentation but yield the CH_2_N(CH_3_)_2_^+^ ion with *m*/*z* = 58 as the lightest, most prevalent fragment, allowing discrimination from linear alkylamines, especially when the alkyl chains are sufficiently long. Subsequent single ion monitoring (SIM) set to *m*/*z* 30, 31, 58, and 60 produced EICs containing information on *n*-alkylamines (not observed), alcohols, tertiary amines (C8–C16), and acid-specific classes, as depicted in Figure S8. The small peaks at *m*/*z* 30 are attributed to alcohols based on retention time, NIST data, and low intensities. The presence of *m*/*z* 58 at the observed elution times for C8 and larger amines strongly suggests that these species are dimethylated tertiary alkylamines. To elucidate the structure of the amines in the sample, LC-ESI-MS/MS experiments were conducted on the water sample and compared to the reference mixture at optimized fragmentation voltages per amine by carbon number. MS/MS spectra of the *n*-alkylamines reference mixture (B, D) and sample (A, C) are presented in Figure S9. The yellow star indicates the parent ion corresponding to the C16 (A, C) and C12 (B, D) amines. The intensity of the fragment ions relative to the parent ion is higher for the primary amine reference compared to the sample. This is another indication of the presence of nonprimary amines in the sample, as these are less prone to CID dissociation resulting from a more rigid structure. The main fragments formed for the *n*-alkylamine standards include *m*/*z* 29, 43, 57, 71, and 85, with *m*/*z* 57 and 71 being the most intense. The *m*/*z* 85 fragment appears to increase with longer chain lengths of the parent ion (comparing A and C with B and D). A charge-remote (or -assisted) driven dissociation mechanism occurs with collision-induced dissociation (CID), resulting in aliphatic fragment ions from hexacyclic hydrogen migrations along the alkyl chain.^45^ Direct comparison of the fragmentation patterns and relative peak intensities for alkylamines with similar carbon chain lengths reveals dissimilarities. These differences arise from architectural discrepancies, implying that B and D are nonprimary, as small *m*/*z* fragments are more probable to form when the longest alkyl chain length is shorter. Validation of the dimethylated nature of the amines in the sample was obtained by spiking to account for any matrix effects on the eventual elution time of the peaks. The EICs of the spiked water sample with 1 ppm tertiary (black) and 1 ppm linear (red) amines are provided in Figure S10, confirming the sole presence of tertiary *N*,*N*-dimethylalkylamines.

### Full Distribution of Alkanes, Alkenes, Oxygenates, and Amines in FT Product Streams

**Figure 6:**
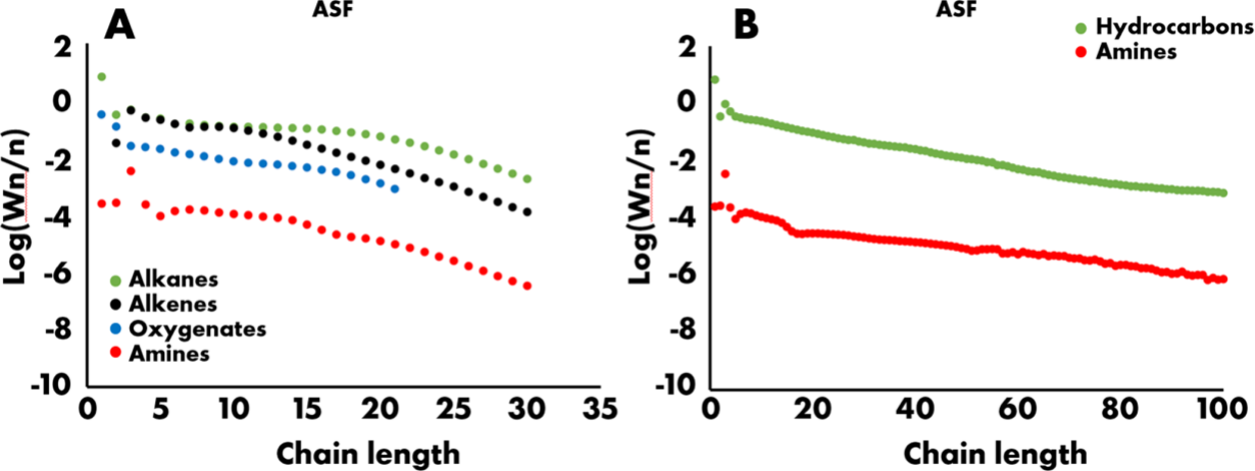
(a) Anderson–Schultz–Flory distribution of alkanes (green), alkenes (black), oxygenates (blue), and amines (red) from the combined product until light wax (b) Anderson–Schultz–Flory distribution of lumped hydrocarbons (green) and experimentally observed amine distribution (red) in the full product.

Detailed analysis of alkanes, alkenes, and oxygenates was done on the light wax sample, oxygenates analysis was done for the water phase, and the online gas analysis provided information on the gaseous alkane and alkene concentrations. The concentrations of the various product classes were combined with the mass flows of each stream to obtain the distributions of alkanes, alkenes, oxygenates, and amines in the FT effluent, as earlier done for the total hydrocarbons. The resulting distributions are provided as the ASF plot in Figure 6a, and averaged concentrations are provided in Table S2. Alkanes are the dominant product class, accounting for 69% of the products in gas, water, and light wax. The alkane distribution shows a straight line between C5 and C15, with deviations on the lighter and heavier sides. Deviations as present in the C4 region are typical for cobalt-based Fischer–Tropsch catalysts and have been ascribed to additional formation of methane on no-growth sites and secondary olefin incorporation in growing chains.^37,38,46^ At the heavier end, the gas/liquid equilibrium in the hot separator (Figure 2A) is the relevant factor. Between C16 and C28, the gas fraction decreases from more than 90% to less than 10%, which is seen as a clear bend in the ASF distribution, as less product is required as light wax. In the total hydrocarbon distribution up to C100, as provided in Figures 2B and 6B, a straight line is obtained in this region. The alkene distribution starts with a low amount of ethylene, ascribed to its high reactivity, matching between C3 and C10 the alkane distribution, and subsequently starts to deviate as secondary reactions become more important.^37−40^ Resultingly, the olefin content in the combined hydrocarbon pool was 27% while dropping from 50% to less than 10% between C10 and C20. The oxygenate contribution amounted to 3.8% in the accounted product and was always below the alkane contribution. The distribution starts with relatively high concentrations of methanol and ethanol and subsequently follows a similar pattern as the alkane product, which is in line with the earlier work of van Steen, who reported on the primary nature of the oxygenate product.^47^ At the same time, it has been reported that secondary reactions can result in decreasing oxygenate concentrations with chain length,^38^ and hence further, studies are warranted to describe the full product slate in the carbon range of C30–C100. The amine product is the least abundant of all, with a total concentration of 0.09% in the accounted product. The distribution is presented in Figure 6A until C30 and in Figure 6B until C100 and is always below the curves of the other products. Specifically, it does not establish the C1 overshoot but rather has an overshoot at C3. Earlier in this paper, we have shown that the amines primarily are *N*,*N*-dimethylalkylamines, and the first product in the homological series of dimethylalkylamines is trimethylamine. Tentatively, we attribute the observed overshoot at the C3 overshoot, rather than at C1, to the preferred formation of *N*,*N*-dimethylamines. The shape of the ASF distribution closely resembles the distribution of oxygenates and alkanes in Figure 6A. In Figure 6B, the total hydrocarbon product and the amine distribution are compared from C1 to C100. A second difference is that around C15, a dip is seen in the amine distribution, and finally, for long products, the hydrocarbon and amine curves get closer to each other. To understand the relation between the hydrocarbon and amine product distribution better and to further substantiate the amine quantifications as described in Figure 3, an attempt was made to relate hydrocarbon and amine distribution mathematically. Details of the procedure are provided in the SI, with model 1 having an unchanged chain growth probability for amines and hydrocarbons, whereas model 2 deploys a slightly higher chain growth probability for amines.

The amine distributions derived with these approaches are shown in Figure S10a and both model 1 and model 2 match well with the measured amine pattern and are acceptable for use. Table S2 and Figure S10b–d provide the experimental and modeled concentrations of amines in the three product phases with a good match for both water and heavy wax of both shape and concentration. In the light wax (Figure S10C), the modeled distributions are very similar in shape versus the experimentally observed distributions, albeit at lower concentrations. The difference between modeled and experimentally observed concentration can be rationalized if the light wax flow is higher than the reported value, and this is supported by the mass balance. From the modeled distributions, it can be concluded that the amine distribution and resulting concentrations in individual product streams can be reasonably well estimated from the hydrocarbon product distribution with a similar growth probability for the amines. Consequently, changes in the selectivity toward the hydrocarbon products can be expected to be reflected through the amine product distribution into the nitrogen balance over water, light wax, and heavy wax. Clear indications for this were obtained from separate other experiments (not shown) where the fraction of nitrogen recovered in the heavy wax stream increased when the C5+ selectivity increased.

## General Discussion

Based on earlier studies, a range of primary alkylamines, amides, or nitriles have been reported as nitrogenates formed during ammonia co-feeding experiments.^25−32^ In our work, although some methylamine was detected, evidence from three independent techniques (Figure 5) showed that most of the product spectrum consisted of tertiary dimethylalkylamines and ammonia. We have found this in other studies at comparable contaminant levels not reported in this paper. The significant ammonia content was not reported in the literature, but it was also not the topic of investigation, and importantly, its presence was not refuted. The presence of ammonia can be rationalized considering that (I) some ammonia is unconverted, (II) disproportionation of methylamine occurs, and/or (III) alkylamine hydrocracking occurs.^48^ Substantially lower trimethylamine contents would be expected for the latter, and disproportionation is unlikely to occur at our operating conditions,^49^ and hence, we ascribe the presence of ammonia to incomplete conversion. The newly observed nitrogenate profile with dimethylamines as the main product was also reflected in a high concentration of C3 amines, as observed by both IC and FIA-SQ-MS, which was not reported in previous publications. Trimethylamine is the first product in the homological series of tertiary amines, and the overshoot could have a similar origin as methane for hydrocarbons.^37,38,46^ An explanation for the different product spectrum in our study is the very low ammonia concentration applied (2.6 ppmV) as compared to 0.5–12 vol %^25−32^ and second the use of a metallic cobalt catalyst versus a cobalt carbide^28,29^ or iron catalyst.^25−27,30−32^ In line with all but one^30^ of the cited studies, a relation was reported between oxygenate and nitrogenate formation. Not only was the presence of nitrogenates reflected in lower oxygenate concentrations,^25−29,31,32^ but a correlation between the types of oxygenate and nitrogenate was also apparent: aldehydes paired with nitriles and alcohols with amines.^26,28^ In our contribution, alcohols were observed as the dominant oxygenate class, which would be in agreement with the formation of amines, but as can be seen in Figure 6a and Table S2, the concentration of amines is too low to have an impact on the alcohol production.

The formation of tertiary amines is an indication of dissociation of ammonia during FT conditions, as otherwise linear amines would be expected as the main product. The dissociation of ammonia on metallic cobalt has been shown by Niemantsverdriet et al. to be facile by a combination of computational and experimental studies, and NH and N species were reported to have higher stability than NH_2_ and NH_3_.^19,20^ Co-feeding studies of Kliger et al. with dimethylamine have shown that the reaction with longer alcohol precursors is similarly possible and results in a homological series of tertiary amines up to C19 with the same chain growth probability for olefins, alcohols, and *N*,*N-*dimethylalkylamines.^31^ In our work, a significant fraction of alcohols was formed with a similar product distribution as amines and alkanes. A speculative potential route toward the observed tertiary amines could be that first dimethylamine fragments are formed from ammonia decomposition products, which subsequently react with alcohol precursors. It needs however to be noted that providing a complete mechanism involving dissociation and chain growth, which also accounts for the formation of byproducts like oxygenates and amines, warrants substantial follow-up work involving both experimental and modeling studies. Potential candidates are DFT,^19,46^ (isotopic) transient kinetics,^29^^29^ and operando XPS, which was recently shown to be able to probe the N speciation over Ru and Fe catalysts during the Haber–Bosch reaction.^50^ The observed similarity between the chain growth probability of amines with oxygenates and alkanes also warrants further attention in light of the long debate on the chain growth mechanism in Fischer–Tropsch synthesis involving either CO or CH_*x*_ insertion.^51^ Another item suggested for follow-up studies is the systematic comparison of the catalytic impact of increasing concentrations of ammonia on catalysts with and without SMSI to establish to what extent the formation of (surface) nitrides depends on the presence of titania overgrowth layers.^52,53^ From the presented results here, we do expect that metallic cobalt is present during Fischer–Tropsch synthesis, but we did not perform controlled oxidation–rereduction experiments to establish the true metal surface area and potential impact on catalysis.^53−55^

Next, we want to discuss the consequence of our findings for potential cost reduction of line-ups to produce SAF from waste and biomass by limiting the gas treatment steps. The ammonia concentration evaluated in our study was close to earlier established NH_3_ contaminant levels in biomass-derived synthesis gas.^12^ In order to evaluate the attractiveness of the cost impact of less extensive treating,^13,15^ three potential consequences need to be discussed: impact on the Fischer–Tropsch section, impact on the hydrocracking section, and impact on the final products; we will treat them in this order. The impact of HCN and NH_3_ on the catalytic performance of cobalt-based catalysts has been evaluated by various groups, and most of the literature studies suggest that this results in a lower activity with no impact on long-term stability.^21^ As the activity can be compensated by the amount of active phase in the reactor, it is important to consider the catalytic selectivity working at the same conversion levels. Selectivity was reported to decrease with a silica system and was found to improve on both alumina- and titania-supported catalysts.^18,23,24^ The potential of selectivity improvement toward longer products as reported by these two groups warrants further study.

SAF requires cracking and isomerization of the raw Fischer–Tropsch product. Cracking and isomerization are done with bifunctional catalysts containing both a metallic and an acidic function. Basic nitrogen compounds interact strongly with the acidic sites and impact the catalytic performance. In oil-based refining, pretreatment can be applied to process oil with >0.25% nitrogen.^56^ The nitrogen content in the combined product in our study was below 10 ppmw, which is much lower than any crude oil. Limited studies are available that evaluate the impact of low concentrations of nitrogen during hydroprocessing. In a study with USY, it was shown that that a variation from 50 to 750 ppmw nitrogen resulted only in a moderate impact on the availability of acidic sites and that activity was in all cases reduced by 2 orders.^57^ In the full nitrogen range, it was shown that the activity could be easily recovered by increasing the reaction temperature. In a study by Ishida, vacuum gasoil was spiked with model compounds to reach concentrations between 0.5 and 100 ppmw. A logarithmic relation between the rate constant and nitrogen concentration was observed, resulting for 10 ppmw feed in roughly 80% activity loss, which could be compensated by increasing the operating temperature by 15–20 °C.^58^ Selectivity toward middle distillates increased due to the presence of nitrogen, and an optimum concentration of 10–15 ppmw was suggested.

Finally, the likelihood and consequence of nitrogen in final products need to be established. Conventional kerosene does not have a specification on nitrogen content,^56^ but for SAF components, the specification is set at 2 ppmw nitrogen in ASTM D7566 and EI 1533, which could impose a blocker. Importantly, it has been established that amines are rapidly converted into ammonia, and in hydrocracking literature, the severity of operation is typically established by calculating the ammonia partial pressure,^57,59^ and hence, no challenge to remain under the specification is expected. To summarize, the evaluation of the consequences of using N-contaminated gas showed no blockers, whereas in both Fischer–Tropsch and hydrocracking catalysis, potential selectivity improvements could be obtained, which makes this a viable option to explore.

## Conclusions

The consequences of ammonia slip in a line-up to produce synthetic aviation fuel from renewable sources were investigated. All effluents of a Fischer–Tropsch experiment with 2.6 ppmV ammonia in the feed were characterized, and the chemical nitrogen concentration was quantified. The nitrogen balance was virtually closed, with 89% recovered in the water, 1% in the light wax, and 7% in the heavy wax stream. The light wax contained the lowest amount of nitrogen, which was established from chain length-dependent hydrophilic–lipophilic balance, resulting in post-condensation separation on polarity. Using three independent analytical techniques, it was proven that tertiary amines were by far the most abundant amine class. Formation of tertiary amines could be rationalized by full ammonia decomposition and the subsequent reaction to dimethylamine, which react with alcohol precursors on the catalyst surface.

Ammonia was predominantly converted into amines during Fischer–Tropsch synthesis, and the product distribution follows the same ASF kinetics as the hydrocarbon products from the naphtha range onward. Despite the low concentrations, the presence of amines up to C120 was established. Using the full-range product distribution, the amine product distribution over the various fractions could be modeled using the same or slightly higher chain growth probability for the amine versus the hydrocarbons. These findings are relevant for mechanistic studies and indicate the good opportunity for cost reduction of a line-up by reducing the treating severity, while more firm validation is required to assess the impact on the hydrocracking section and the final products.

## Supporting Information Available

List of characterization methods; DI-ESI-TOFMS results of all samples; MALDI-FT-ICR-MS of heavy wax with DMHCA and DHB; amine oxidation patterns; alcohols and amines partitioning over light wax and water; extra information on amine speciation with extracted ion chromatograms; and experimental and modeled nitrogen concentrations in all streams (PDF)
