# Supplementary material for: Fischer–Tropsch Synthesis for the Production of Sustainable Aviation Fuel: Formation of Tertiary Amines from Ammonia Contaminants
Source: ACS Omega. 2024 Jul 10;9(29):31974–85. doi: 10.1021/acsomega.4c03734 (PMC11270693; doi:10.1021/acsomega.4c03734)
Supplement: Supplementary file 1 — ao4c03734_si_001.pdf [file ao4c03734_si_001.pdf]

SUPPORTING INFORMATION

FISCHER-TROPSCH SYNTHESIS FOR THE PRODUCTION OF SUSTAINABLE AVIATION FUEL: FORMATION OF TERTIARY AMINES FROM AMMONIA CONTAMINANTS.

Robert L. C. Voeten[1§], Floran Hendriks[1§], G. Leendert Bezemer[1*]

1 Energy Transition Campus Amsterdam, Shell Global Solutions International B.V., Grasweg 31, 1031 HW Amsterdam, the Netherlands

§ R. V. and F.H. contributed equally to this paper

* To whom correspondence should be addressed: Leendert.Bezemer@shell.com

Section 1 Experimental details

Total nitrogen content Chemiluminescence with a Thermo Analyzer TN3000 was used for measurement of water, light wax and heavy wax samples. For liquid samples the ASTM D4629 protocol was used, while for heavy wax samples a new protocol was developed, adapted from ASTM D5762. About 5 mg of the sample was inserted in a platinum crucible and transferred to the cup quartz boat. The sample was gradually inserted into a combustion tube of 1050 °C where it oxidized in an oxygen atmosphere. Combustion products were reduced to NO over a molybdenum converter. Subsequently the NO formed is converted into excited nitrogen oxide (NO_2*). The light emitted from decay of excited NO_2* was detected by a photomultiplier tube. The resulting signal is a measure for the nitrogen concentration in the specimen. Results are reported with 20% confidence limit from detection limit of 0.5 ppmw onwards. For water and light wax samples, liquid injection can be used with higher precision resulting in a detection limit of 0.1 ppmw and 10% confidence limit.

IC-CD A Thermo Scientific IC-5000 system consisting of a pump with eluent generation (Dionex EGC 500 MSA), electrical ion suppression, and equipped with a Thermo Scientific CS10 column (2 mm x 250 mm) thermostatic at 30 ºC was used for IC-CD analyses. Eluent flow rate was 1 mL/min 20 mM methane sulfonic acid. Suppressed conductivity detection was performed with 59 mA through the electronic suppressor, with a sampling frequency of 5 Hz. Ten µL of mixed calibration standards (ammonium, methyl amine, dimethyl amine, trimethyl amine and n-propyl amine; concentration ranged from 10 ppb (w/v) to 30 ppm (w/v) in water) were injected. Calibration lines were constructed by quadratic curve fitting for the area vs. concentration. All water samples were diluted 20x in water prior to injection.

FIA-ESI-sQMS Flow injection analysis (FIA) was conducted using an Agilent 1290 Infinity II quaternary pump and autosampler coupled to an Agilent MSD XT (6135) single quadrupole mass spectrometer (sQMS) using electrospray ionization (ESI) in positive mode. To optimize for low m/z detection the following parameters were changed from the autotuned value: octopole radiofrequency voltage: 125 V, fragmentor voltage: 80 V, and skimmer voltage: 20 V. A restriction capillary was installed between the autosampler and MS to ensure adequate backpressure for effective pumping by the quaternary pump. For water samples, the eluent comprised milli-Q water with 0.1% (v/v) formic acid (FA). The initial analysis was performed after diluting the sample 10-fold in eluent. The standard addition conditions were calculated based on the results obtained from the initial analysis. A mixture of n-alkyl amines (C1, C2, C3, C5, C6, and C8) was prepared to match the anticipated levels in the sample, with five addition points. The addition points were selected so that five points would range from 0 addition to 1-1.5 times the expected concentration. Data processing involved generating extracted ion currents for each amine, assuming all amines to be n-alkyl amines within a range of ±0.5 Da. The area under the curve for each peak was measured. For the components in the amine mix, the areas were used to generate standard addition curves. The amount of amine in the samples was established by back calculating the intercept on y=0 and adjusting for sample dilution. For the amines that were not in the mix the average of the two closest response factors was determined and used. For light wax samples, the same setup was utilized, but with a different eluent consisting of 50/50 methanol in toluene with 0.1% (v/v) formic acid for both flow injection analysis and sample preparation.

ESI-TOFMS and ESI-/MALDI-FT-ICR-MS The received samples consisted of three types: I) water-based, II) light wax-based, and III) Heavy Wax-based. Various sample preparation methods were explored, and the most promising ones for ESI-MS experiments were as follows: I) The sample was dissolved in a mixture of water and MeOH at a ratio of 1:100, with the addition of 0.1% FA. II) A liquid-liquid extraction (LLE) was performed using a mixture of water and MeOH at a 1:1 ratio, along with 0.1% FA. The water layer obtained from the extraction was subsequently diluted 1x in a solution of water and MeOH with 0.1% FA) III) The sample was ground to a fine powder using a mortar and then dissolved in toluene at 80°C to obtain a solution with a concentration of 2 mg/mL. MeOH was added in a 1:1 volume/volume ratio, resulting in a 1 mg/mL solution. Finally, 0.1% volume of FA was added. For MALDI experiments, 10 mg wax is grinded with mortar and pestle. The fine wax powder is further mixed-grinded with 10 mg DHB (note that DMHCA yielded no amine distribution) for optimal sensitivity (1:5 and 1:10 yielded less satisfactory results). The mixed powders are applied and smeared on a MALDI spot with a spatula to form a thin layer. Ideally, the powder is homogenously distributed across the spot, but this is not always possible as the mix does not spread easily (inherent to the wax). To ensure representative sampling, the laser shots are fired with a random walk model across the spot. Unless stated otherwise, all samples were directly infused at a flow rate of 10 µL/min and analyzed in positive ESI mode. The measurements were performed using either a Synapt-G2 Si Time of Flight Mass Spectrometer (TOFMS) system (Waters) or a high-resolution 12T Bruker solariX XR FT-ICR-MS (Bruker Daltonics, Bremen, Germany) equipped with a ParaCell and an ESI source. Data collection was conducted over a 2-minute period. The ESI-TOFMS spectra were centroided and processed in Elemental Composition software for molecular formula annotation. The FT-ICR-MS spectra were processed using DataAnalysis 5.3 software (Bruker Daltonics), exported as csv files, and imported into Composer software for molecular formula annotation. In both cases, the annotations were transferred to Excel (Microsoft 365 Version 2208 64-bit) for the visualization and analysis of amine distributions by plotting relative intensities or concentrations against carbon number.

LC-ESI-TOFMS Liquid chromatography (LC) separations were performed using a Waters Acquity UPLC system with QTOF-MS detection in resolution mode, equipped with a quaternary pump. Separation was achieved using a Kinetex 1.7 µm BEH C8 column (100x2.1 mm). The LC system utilized a gradient of water (A) and acetonitrile (ACN; B) to elute the analytes. The gradient started at a composition of 95% water and 5% acetonitrile for 1 minute, followed by a ramp to 5% water and 95% acetonitrile over 14 minutes. The composition was then maintained isocratically for 13 minutes before being adjusted back to 95% water and 5% acetonitrile in 2 minutes. An injection volume (Vinj) of 5 µL and a flow rate of 0.4 mL/min were

employed. Prior to analysis, a standard amine mix was prepared, which included C5, C6, C8, C10, C12, C16, C18, and C22 at approximately 100 ppm concentration in a mixture of methanol (MeOH), toluene (Tol), and formic acid (FA) in a ratio of 50/50/0.1. The stock solution was diluted 100 times before being injected into the LC system.

SPME-GCMS The water samples were subjected to headspace solid-phase microextraction (HS-SPME) at a temperature of 60°C, both on the liquid and in the headspace. In both cases, a multipurpose PMDS/DVB/CWR fiber was employed for extraction. The analytes obtained from the water sample were injected into the gas chromatography-mass spectrometry (GC-MS) system using a splitless injection technique. The GC system was equipped with an SPSil 5CB column measuring 50m x 0.32mm x 1.2µm. The initial temperature of the column was set at 40°C for 1 minute and then increased at a rate of 25°C per minute for a duration of 10 minutes until reaching 325°C. Helium was used as the carrier gas with a flow rate of 2.0 ml/min. The mass selective detector source temperature was maintained at 230°C. To extract structural information and facilitate identification, the fragmentation patterns of the eluting peaks were analyzed. This information was then utilized to identify and annotate relevant components in the chromatogram. Additionally, single ion monitoring was employed to enhance sensitivity and selectivity for specific compound classes of interest, such as primary, secondary, and tertiary amines and alcohols and acids.

Modelling amine distribution Starting from the total hydrocarbon product distribution two models were made to derive from them an amine distribution. The first model assumes I) the observed experimentally split between gas, light wax and heavy wax as the basis for the amine partitioning with water/light wax split governed by the HLB, II) Light amines are adjusted to fit the observations: C1 is 90% reduced, C2 is doubled and C3 is 8.7 times increased versus hydrocarbons, III) Amines have the same chain growth probability compared to hydrocarbons, and IV) preservation of nitrogen balance holds. The second model deviates from the third assumption of model 1 by assuming that amines have a slightly higher chain growth probability compared to hydrocarbons.

Section 2 Water phase characterization

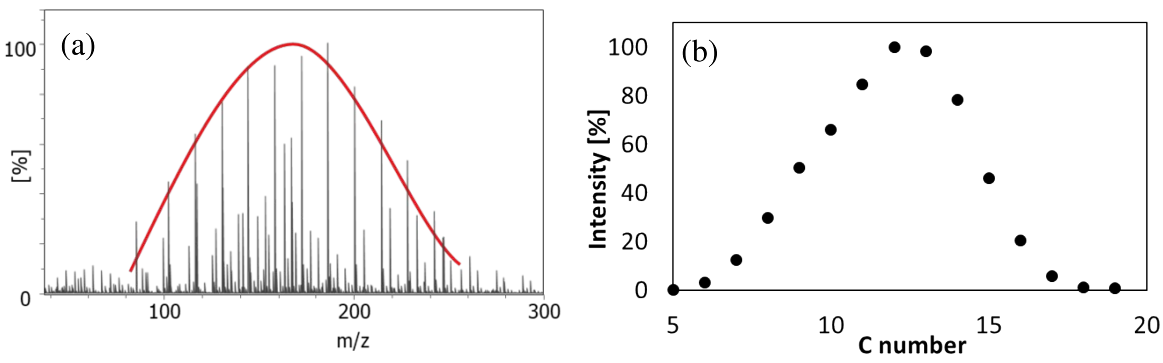

Figure S1 (a) Mass spectrum obtained for the water sample diluted 1:100 vol:vol with MQ/MeOH containing 0.1% FA measured with direct infusion ESI-TOFMS and (b) corresponding relative amine content distribution in the water sample.

Table S1 Overview of concentrations in the water sample of ammonia as measured with Ion Chromatography (IC) and amines measured with Ion Chromatography, Single Quadrupole MS (SQMS) and ESI-TOFMS and consolidated results. All concentrations are expressed in ppmw N.

	IC	SQMS	Extrapolated	ESI-TOF-MS	Consolidated
**Ammonia**	17.9				17.9
**C1**	0.8	0.0			0.8
**C2**	1.1	0.2			1.1
**C3**	16.6	13.1			16.6
**C4**		1.2			1.2
**C5**		0.5		0.0	0.5
**C6**		0.8		0.0	0.8
**C7**		0.9		0.1	0.9
**C8**		0.8		0.1	0.8
**C9**		0.7		0.3	0.7
**C10**		0.5	0.7	0.3	0.7
**C11**		0.3	0.6	0.4	0.6
**C12**		0.2	0.5	0.5	0.5
**C13**		0.1	0.5	0.5	0.5
**C14**				0.4	0.4
**C15**				0.2	0.2
**C16**				0.1	0.1
**C17**				0.0	0.0
**C18**				0.0	0.0
**C19**				0.0	0.0
**Total**					44.4

Section 3 Heavy Wax characterization

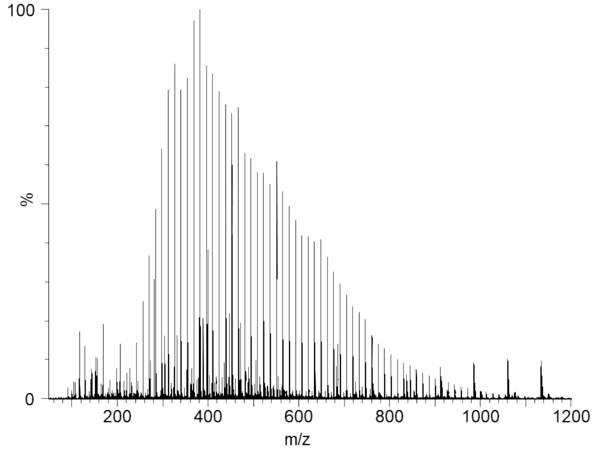

Figure S2 Mass spectrum obtained with DI-ESI-TOFMS for the heavy wax sample dissolved in toluene/methanol (50/50) containing 0.1% FA.

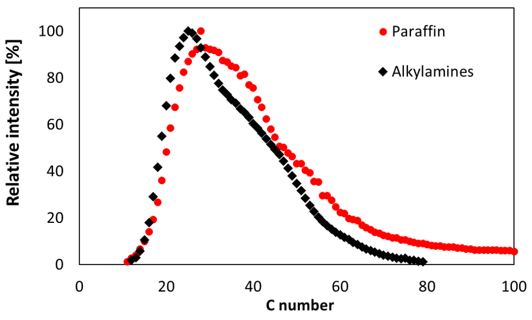

Figure S3 Overlay of relative distribution profiles of alkylamines in the heavy wax sample as observed by DI-ESI-TOFMS after dissolution in toluene/methanol (50/50) containing 0.1% FA (black) and paraffins as observed by GC-FID (red).

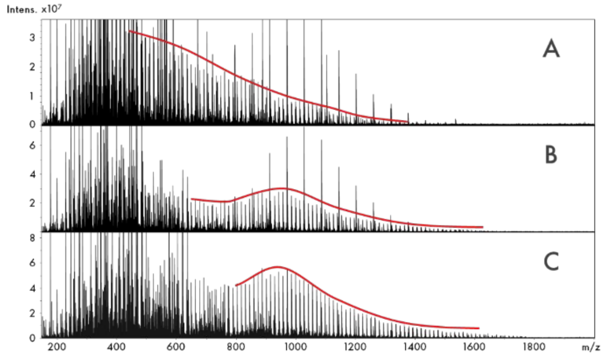

Figure S4 Mass spectra obtained for the heavy wax sample mixed with DHB (A, 1:1; B, 1:5; and C, 1:10 mass:mass) during MALDI-FT-ICR-MS. The red line indicates the amine distribution.

Section 4 Light wax characterization

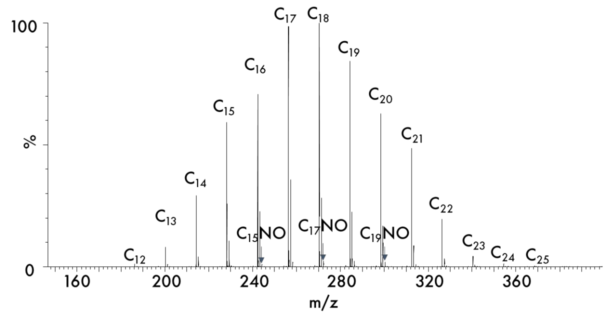

Figure S5 Mass spectrum measured with DI-ESI-TOFMS of light wax sample after Liquid-Liquid Extraction (1:1 vol:vol with MQ water/methanol containing 0.1% FA).

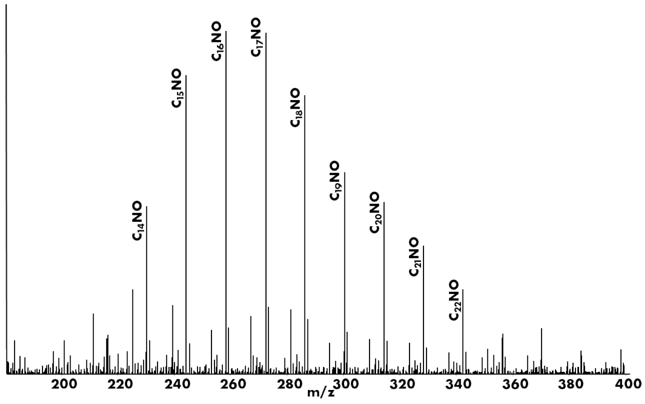

Figure S6 Summation of single quadrupole mass spectra with Flow Injection Analysis using toluene/methanol on the light wax sample indicating a distinct CH_2 distribution ($\Delta m/z = 14$) differing from the alkylamine series by a m/z of 16.

Section 5 Amine speciation

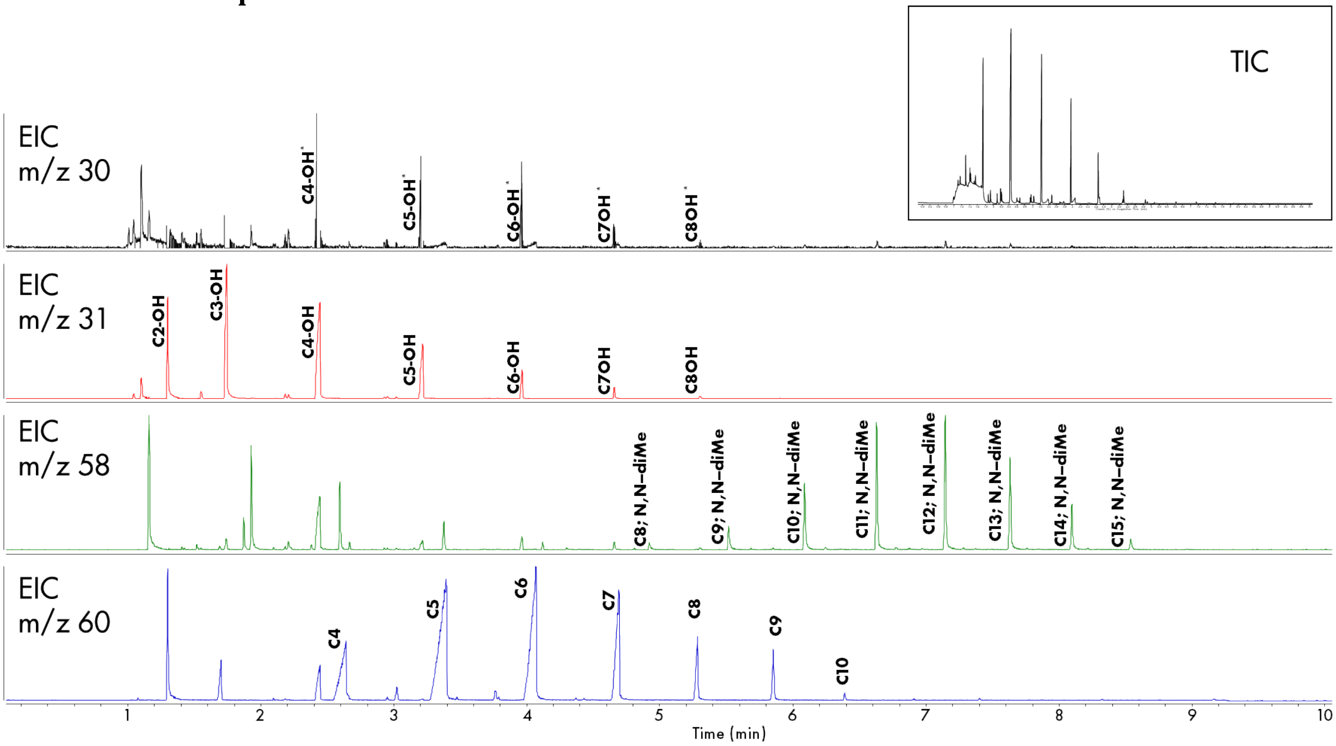

Figure S7 Extracted ion chromatograms of m/z 30 (black), 31 (red), 58 (green), and 60 (blue) observed from the water fraction by GC-EI-MS in single ion monitoring (SIM)-mode. The EICs respectively regard fragments specific for primary amines (not observed), alcohols, dimethylated amines (i.e., tertiary/branched amines), and acids. The peaks observed at m/z 30 and marked with * are attributed to alcohols based on retention time, NIST data and intensity. The insert shows the TIC chromatogram.

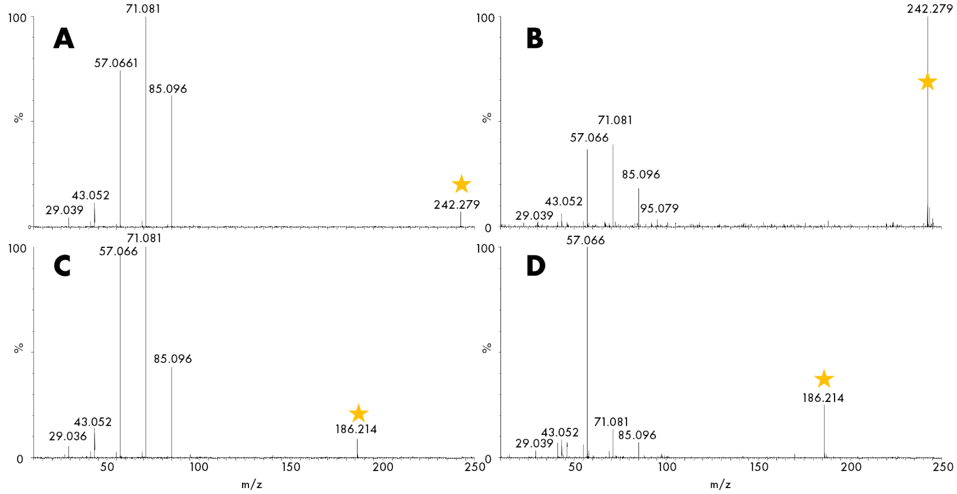

Figure S8 MS/MS fragmentation spectra of C16 and C12 primary linear amines in (A, C) 1 ppm reference solution and (B, D) the water sample. The respective parent ions are indicated by yellow stars. The different fragmentation patterns with same parent ion mass are indicative of structural differences.

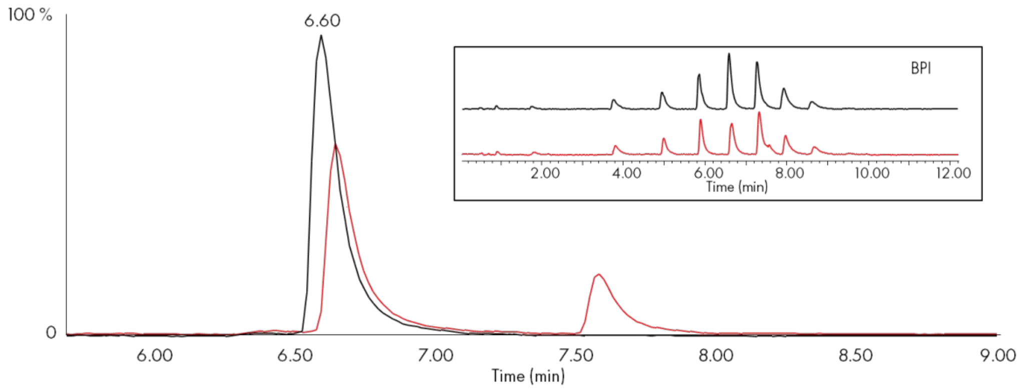

Figure S9 Overlay of EICs of m/z 188.222 for the water sample spiked with 1 ppm dodecylamine (Red) and spiked with 1 ppm *N,N*-dimethyldecylamine (Black). The *N,N*-dimethyldecylamine coelutes with the peak of the water sample (6.60 min), the dodecylamine clearly elutes at a later time (7.59 min) from the column, validating the dimethylated nature of the amine in the water sample. The insert shows the BPI chromatogram for both spiking experiments.

Section 6 Full description of amine product distribution

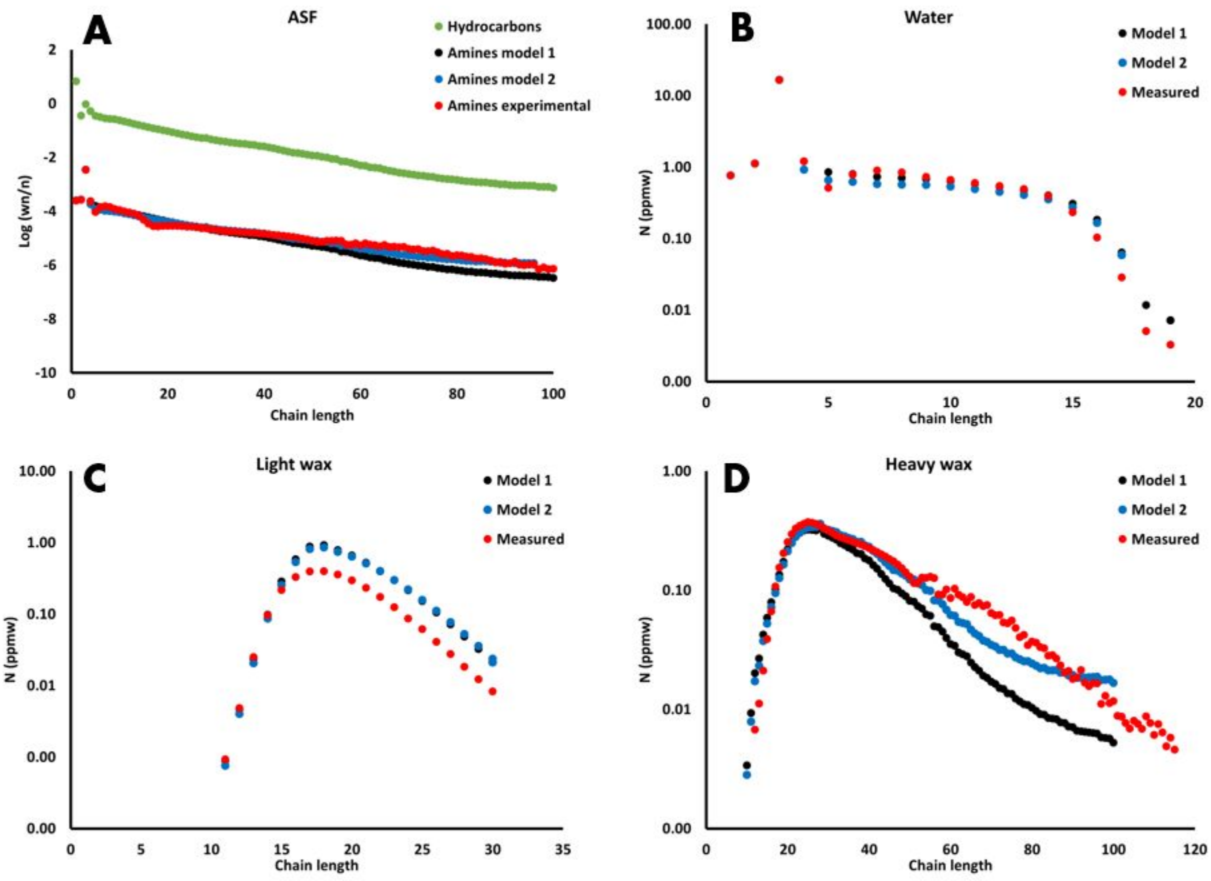

Figure S10: (a) Anderson-Schultz-Flory distribution of total hydrocarbons (green) and experimentally observed amine distribution (red). The amine distribution derived from the hydrocarbon distribution using unchanged (black) or increased (blue) chain growth probability of amines. (b) Experimentally observed and distribution derived from ASF and HLB partitioning of amines in (b) water, (c) light wax and (d) heavy wax.

Table S2: Amine concentration in water, light wax and heavy wax as experimentally established and derived with model 1 and model 2 from the hydrocarbon product distribution. Values are expressed in ppmw N.

	Water	Light wax	Heavy wax
**Experimental**	26.5	2.9	11.6
**Model 1**	26.6	6.1	9.1
**Model 2**	25.1	5.8	12.8

Table S3: Paraffin, Olefin, Oxygenate and Amine concentration (wt%) in the various outlet streams

	Paraffin	Olefin	Oxygenate	Amines
**Gas**	74.9	25.1	ND	ND
**Water**	ND	ND	0.59	0.015
**Light wax**	69.2	27.3	3.5	0.006
**Combined up to light wax**	69.3	26.9	3.8	0.090
**Heavy wax**	100 (combined hydrocarbons)			0.049
